# DNA Methylation Mediates EMT Gene Expression in Human Pancreatic Ductal Adenocarcinoma Cell Lines

**DOI:** 10.3390/ijms23042117

**Published:** 2022-02-14

**Authors:** Maria Urbanova, Verona Buocikova, Lenka Trnkova, Sabina Strapcova, Viera Horvathova Kajabova, Emma Barreto Melian, Maria Novisedlakova, Miroslav Tomas, Peter Dubovan, Julie Earl, Jozef Bizik, Eliska Svastova, Sona Ciernikova, Bozena Smolkova

**Affiliations:** 1 Department of Molecular Oncology, Cancer Research Institute, Biomedical Research Center, Slovak Academy of Sciences, Dubravska Cesta 9, 845 05 Bratislava, Slovakia; maria.urbanova@savba.sk (M.U.); verona.buocikova@savba.sk (V.B.); lenka.trnkova@savba.sk (L.T.); viera.kajabova@savba.sk (V.H.K.); miroslav.tomas@nou.sk (M.T.); peter.dubovan@nou.sk (P.D.); jozef.bizik@savba.sk (J.B.); sona.ciernikova@savba.sk (S.C.); 2 Department of Tumor Biology, Institute of Virology, Biomedical Research Center, Slovak Academy of Sciences, Dubravska Cesta 9, 845 05 Bratislava, Slovakia; sabina.strapcova@savba.sk (S.S.); eliska.svastova@savba.sk (E.S.); 3 Molecular Epidemiology and Predictive Tumor Markers Group, Ramón y Cajal Health Research Institute (IRYCIS), Biomedical Research Network in Cancer (CIBERONC), Carretera Colmenar Km 9,100, 28034 Madrid, Spain; emma.barreto@salud.madrid.org (E.B.M.); julie.earl@live.co.uk (J.E.); 4 Oncology Outpatient Clinic, Hospital of the Hospitaller Order of Saint John of God, 814 65 Bratislava, Slovakia; mnovisedlakova@milosrdni.sk; 5 Department of Surgical Oncology, National Cancer Institute, Slovak Medical University, Klenova 1, 833 10 Bratislava, Slovakia

**Keywords:** PDAC, inflammation, hypoxia, epithelial-to-mesenchymal transition, DNA methylation

## Abstract

Due to abundant stroma and extracellular matrix, accompanied by lack of vascularization, pancreatic ductal adenocarcinoma (PDAC) is characterized by severe hypoxia. Epigenetic regulation is likely one of the mechanisms driving hypoxia-induced epithelial-to-mesenchymal transition (EMT), responsible for PDAC aggressiveness and dismal prognosis. To verify the role of DNA methylation in this process, we assessed gene expression and DNA methylation changes in four PDAC cell lines. BxPC-3, MIA PaCa-2, PANC-1, and SU.86.86 cells were exposed to conditioned media containing cytokines and inflammatory molecules in normoxic and hypoxic (1% O_2_) conditions for 2 and 6 days. Cancer Inflammation and Immunity Crosstalk and Human Epithelial to Mesenchymal Transition RT² Profiler PCR Arrays were used to identify top deregulated inflammatory and EMT-related genes. Their mRNA expression and DNA methylation were quantified by qRT-PCR and pyrosequencing. BxPC-3 and SU.86.86 cell lines were the most sensitive to hypoxia and inflammation. Although the methylation of gene promoters correlated with gene expression negatively, it was not significantly influenced by experimental conditions. However, DNA methyltransferase inhibitor decitabine efficiently decreased DNA methylation up to 53% and reactivated all silenced genes. These results confirm the role of DNA methylation in EMT-related gene regulation and uncover possible new targets involved in PDAC progression.

## 1. Introduction

Pancreatic ductal adenocarcinoma (PDAC), representing more than 90% of all pancreatic cancers, is estimated to become the second leading cause of cancer-related deaths in developed countries by 2030 [1]. Patient prognosis is mainly affected by the time of disease diagnostics. However, only 11% of PDACs are detected early, with a 5-year survival rate of 39%. If cancer has spread to surrounding tissues or organs, a 5-year survival rate drops down to 13%, and for the 52% of patients diagnosed in the late stage, it decreases to only 3% [2]. High PDAC mortality is also a consequence of its aggressive nature with early local invasion and resistance to conventional treatment.

Accumulation of genetic, epigenetic, and morphological changes in pancreatic ductal cells is causal in disease initiation and development. The progression from hyperplasia through dysplasia to invasive PDAC is at the molecular level associated with telomere shortening and accumulation of mutations in *KRAS*, *ERBB2*, *CDKN2A* in low-grade, and *TP53*, *SMAD4*, and *BRCA2* in high-grade pre-invasive precursor lesions [3]. However, the mutational burden is not enough to comprehensively explain PDAC pathogenesis. Integration of genomic and epigenomic data demonstrates that mutational alteration in oncogenes, such as *KRAS*, induces downstream signaling leading to direct regulation of histone proteins as well as histone and DNA-modifying enzymes [4]. While genetics is critical for PDAC initiation and early progression, the acquisition of tumor heterogeneity is associated with specific epigenomic landscapes [5,6].

Besides cell-intrinsic (mutation background, epigenetic state), several extrinsic factors in the tumor microenvironment, such as inflammation, hypoxia with related oxidative stress, and acidosis, significantly contribute to PDAC aggressiveness [7,8]. The fibro-inflammatory stroma of chronic pancreatitis resembles that of pancreatic cancer, and aberrant inflammatory signaling contributes to the malignant transformation of pancreatic cells [9,10]. On the other hand, hypoxia is one of the main players inducing metastatic cascade: tumor cell intravasation, migration, survival in the bloodstream, extravasation, and colonization [11]. The hypoxic PDAC microenvironment resides from poor vascularization and rapid proliferation of cancer cells. The presence of hypoxic areas in the tumors correlates with a worse prognosis [12]. Tumor cells develop efficient adaptive metabolic strategies in hypoxic conditions to satisfy their high energetic demands. Hypoxia-inducible factors (HIFs) are activating transcriptional factors (TFs), securing the physiological response of cancer cells to hypoxia by stimulation of genes involved in angiogenesis and glycolysis [13]. We and others have provided evidence that hypoxia is accompanied by HIF1 induction in various cancers, including PDAC [14,15,16]. Intratumoral hypoxia mediates epithelial to mesenchymal transition (EMT), whose major inducer is transforming growth factor-β (TGF-β) along with cytokines and growth factors secreted by the tumor microenvironment [17,18]. EMT results in loss of cell adhesion, abnormal apical-basal polarity, and cytoskeletal reorganization, which raises tumor cell motility, invasiveness, and stemness [19]. Mesenchymal phenotype increases resistance to apoptosis and elevates the production of extracellular matrix (ECM) components by activated pancreatic stellate cells. One of the main EMT features is the functional loss of E-cadherin expression [20]. Hypoxia-induced pathways critically contribute to the deregulation of EMT-TFs, including SNAIl, TWIST1, ZEB1, ZEB2, or SIP1 [21]. These TFs, promoting cell polarity loss by destroying tight junctions and degrading adhesion molecules, were detected to be overexpressed in PDAC [22]. The tumors’ hypoxic environment can induce EMT by reducing the activity of ten-eleven translocation (TET) enzymes, which are essential in the process of cytosine demethylation, and bind the oxygen molecule as an essential cofactor [23].

Accumulating evidence reveals that hypoxia in cancers directly influences chromatin remodeling events like DNA methylation and histone modifications [24]. Epigenetic regulation is also implicated in dynamic changes underlying metastable or stable EMT transitions [25]. However, the study of epigenetic changes under hypoxic conditions is at the beginning in PDAC, with better-characterized microRNA and long non-coding RNA regulation, post-translational modifications of histones, and expression of epigenetic regulator proteins [26]. SNAI1 can recruit multiple chromatin-modifying enzymes, including LSD1, HDAC1, HDAC2, PRC2, and others to the E-cadherin promoter, inducing DNMT-mediated DNA methylation [27,28]. Earlier studies demonstrated that downregulation of E-cadherin in metastatic PDAC cells was guided by a SNAI1/HDAC1/HDAC2 repressor complex [29]. Importantly, PDAC models and human samples confirmed these findings. It is generally accepted that there is a global increase in DNA methylation after a period of hypoxia, which can be at least in part attributable to HIF-mediated expression of histone-modifying enzymes [30].

Given the limited options for PDAC treatment and the suggested role of DNA methylation in cancer treatment resistance, understanding epigenetic mechanisms underlying PDAC invasiveness and metastasis makes it possible to identify new therapeutic targets [31]. Herein, we examined the extent to which epigenetic regulation influences gene expression of EMT-related genes in PDAC. Particularly, the role of DNA methylation in the inflammation- and hypoxia-driven EMT model has been investigated in a subset of PDAC cell lines.

## 2. Results

Four PDAC cell lines, BxPC-3, MIA PaCa-2, and PANC-1, derived from the primary adenocarcinoma, and SU86.86 cells derived from liver metastasis, were used to assess EMT-related changes in gene expression and DNA methylation. Inflammatory conditions were modeled by indirect cell co-cultivation through a conditioned media (CM) containing a wide range of cytokines and inflammatory molecules produced by activated fibroblasts [32]. Cells were cultured in a monolayer for two and six days in either DMEM under normoxic conditions (Control), CM in normoxia (CM), DMEM in hypoxia (1% O_2_, HY), or CM in hypoxia (CM + HY) (for details, see Material and Methods).

### 2.1. Inflammation and Hypoxia-Mediated Gene Expression Changes after 2-Day Exposure

**Figure 1:**
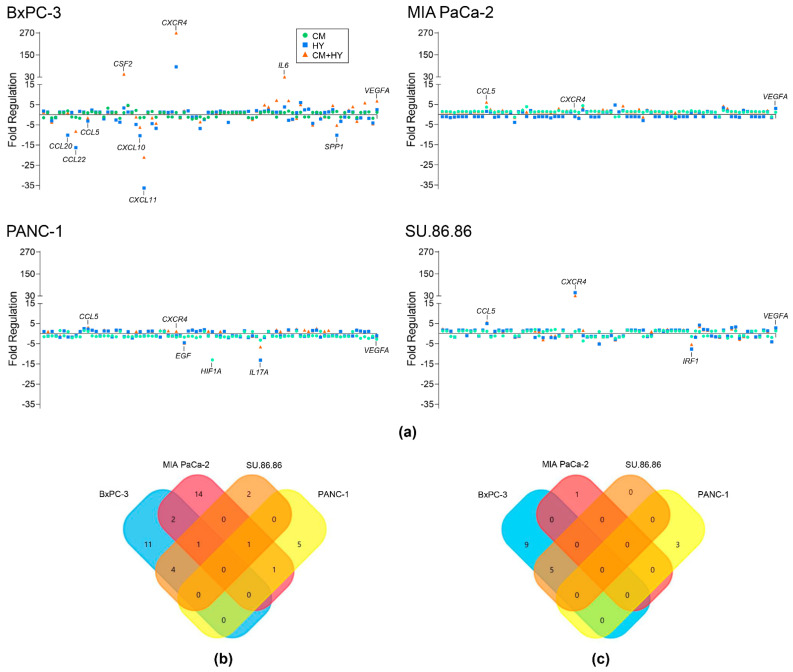
Inflammatory gene expression changes after 2-day cultivation of cells in inflammatory (CM), hypoxic (HY) conditions, and their combination (CM + HY) measured using Cancer Inflammation and Immunity Crosstalk RT^2^ Profiler PCR Array (**a**). Gene expression changes are plotted as fold regulation values (−1/fold change for FC below 1); (**b**) Venn diagram showing overlapping genes with more than two-fold upregulation by CM + HY exposure; (**c**) Venn diagram showing overlapping genes with more than two-fold downregulation by CM + HY exposure. CM, conditioned media; HY, 1% hypoxia.

To identify inflammation- and hypoxia-induced EMT-related gene expression changes, we used Cancer Inflammation and Immunity Crosstalk RT² Profiler and Human Epithelial to Mesenchymal Transition RT² Profiler PCR Arrays (PAHS-181Z and PAHS-090ZA, respectively). Each of them allowed us to analyze 84 genes or biological pathways, either mediating communication between tumor cells and the cellular mediators of inflammation and immunity or tumor metastasis, stem cell differentiation, and development. A two-fold change (FC) was set as a cut-off for upregulation and 0.5 for downregulation. An example of an inflammatory factor present in CM is CXCL12 (13.7% increase in CM over DMEM, unpublished data), a ligand for the C-X-C motif chemokine receptor 4 (CXCR4). Increased content of proinflammatory IL-1α in the CM (10.3%, unpublished data), which constitutively activates the NF-κB signaling pathway, could influence the expression of other inflammatory genes such as vascular endothelial growth factor A (*VEGFA)*. However, only a few inflammatory genes showed more than two-fold change after cultivating cells in CM alone (Figure 1a). *VEGFA* upregulation by HY and CM + HY was found in all cell lines except for PANC-1. The highest upregulation of the C-C motif chemokine ligand 5 (*CCL5*) gene was induced by CM + HY. With the upregulation of 18 (Figure 1b) and downregulation of 14 genes (Figure 1c), the BxPC-3 cell line was the most sensitive to CM + HY exposure. Although 19 inflammatory genes were upregulated by more than two-fold in the MIA PaCa-2 cell line, the magnitude of these changes was lower, and only one was downregulated below 0.5-fold. The PANC-1 and SU.86.86 cell lines were more resistant to studied factors with upregulation of only a small number of the analyzed genes (7 and 8, respectively), while downregulation below 0.5-fold was observed only in 3 and 5 genes in these cell lines, respectively. In general, hypoxia was a more potent factor in inducing an inflammatory response in cells. The combination of CM + HY had the most pronounced effect on changes in inflammatory gene expression, with the *CXCR4* receptor gene being among the top upregulated in all cell lines. The top downregulated gene was *CCL11* by HY in BxPC-3 cells.

**Figure 2:**
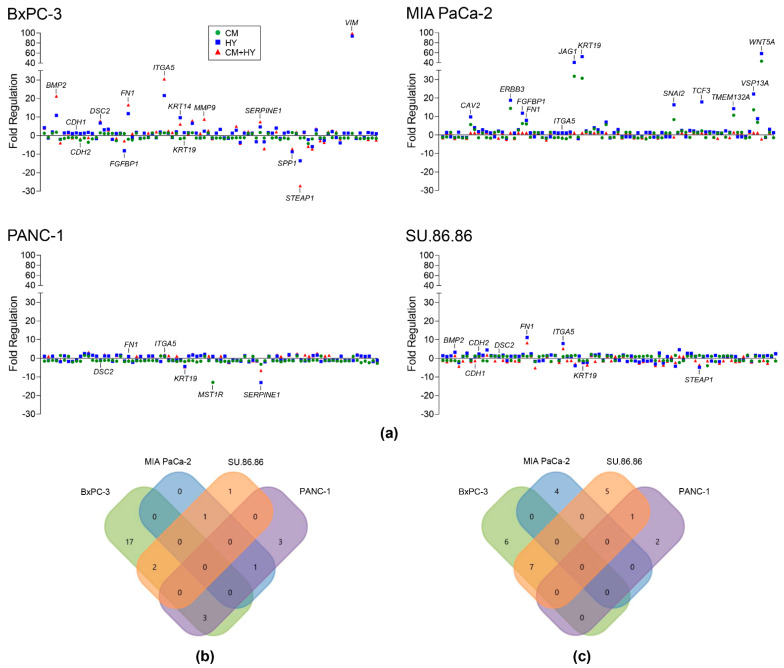
EMT gene expression changes after 2-days cultivation of cells in inflammatory (CM), hypoxic (HY) conditions and their combination (CM + HY) measured using Human Epithelial to Mesenchymal Transition RT² Profiler PCR Array (**a**). Gene expression changes are plotted as fold regulation values (−1/fold change for FC below 1); (**b**) Venn diagram showing overlapping genes with more than two-fold upregulation by CM + HY exposure; (**c**) Venn diagram showing overlapping genes with more than two-fold downregulation by CM + HY exposure. CM, conditioned media; HY, 1% hypoxia.

Extensive changes were found in the expression levels of EMT-related genes, although their magnitude was lower than for inflammatory genes (Figure 2a). In line with previous results, showing 17 and 22 upregulated and 10 and 13 downregulated genes by HY and CM + HY, respectively, the BxPC-3 cells were the most sensitive to studied factors (Figure 2b,c). Although the top-upregulated *VIM* gene showed an almost 100-fold change in BxPC-3 cells due to HY, no changes in *VIM* expression were found in the other cell lines. The top downregulated gene, *STEAP1*, with fold regulation value −27.0, was identified in the same cell line. CM-induced gene expression changes were milder, except for MIA PaCa-2 with 16 upregulated and 2 downregulated genes. However, only 2 genes were upregulated and 4 downregulated in this cell line by a combination of CM + HY. Interestingly, some of the genes upregulated in MIA PaCa-2 were downregulated in other cell lines, e.g., *KRT19*. The most resistant to all experimental conditions were PANC-1 cells, where CM + HY only upregulated 7 and downregulated 3 genes.

Based on these findings and published literature, three inflammatory genes (*CCL5*, *CXCR4*, *VEGFA*), five EMT-TFs (*SNAI1*, *SNAI2*, *ZEB1*, *ZEB2*, *TWIST1*), and 12 EMT-related genes (*CDH1*, *KRT19*, *OCLN*, *STEAP1*, *TSPAN13*, *CDH2*, *FN1*, *ITGA5*, *BMP2*, *NID2*, *SERPINE1*, and *DSC2*), were selected for validation/analysis by qRT-PCR. Genotyping assays and primer sequences are listed in Materials and Methods and Tables S1 and S2.

**Figure 3:**
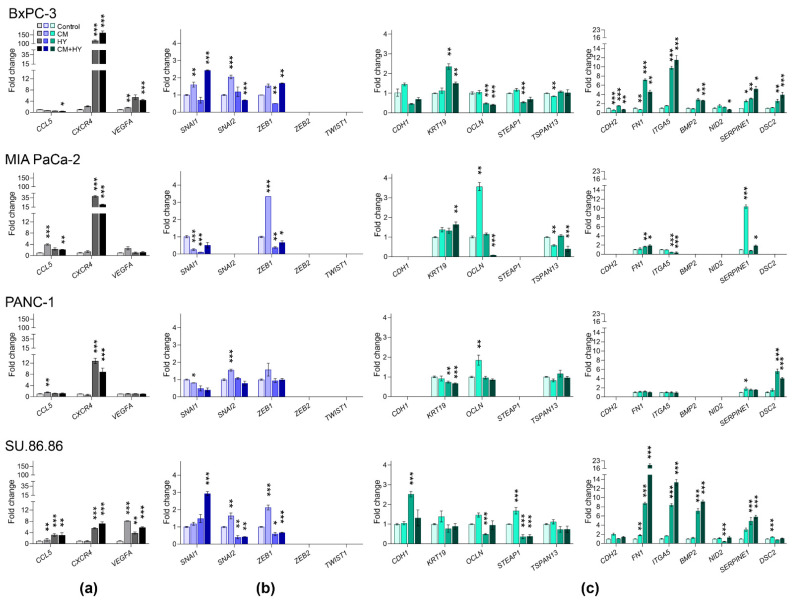
Changes in the expression of (**a**) inflammatory, (**b**) EMT-TFs, and (**c**) EMT-related genes after 2-day cell cultivation in inflammatory (CM), hypoxic (HY) conditions, and their combination (CM + HY), relative to Control. Inflammatory genes are highlighted by grey, EMT-TFs by blue, and EMT-related genes by green color; CM, conditioned media; HY, 1% hypoxia; * *p* < 0.05, ** *p* < 0.01, *** *p* < 0.001.

Gene expression changes after 2-day exposure to experimental conditions (CM, HY and CM + HY) are provided in Figure 3 and Table S3. In agreement with previous findings, *CXCR4* was upregulated by HY and CM + HY in all studied cell lines. A significant upregulation of *VEGFA* gene expression was observed due to CM and CM + HY in BxPC-3 cells and all studied combinations in SU.86.86 cells. MIA PaCa-2 and PANC-1 showed to be relatively resistant to all treatment conditions. In line with the data from the PCR array, the *CCL5* gene was downregulated in BxPC-3 cells, while its upregulation was observed in MIA PaCa-2, PANC-1, and SU.86.86 cell lines.

Gene expression of the EMT-TFs was affected moderately, with most changes below two-fold, except for *SNAI1* by CM + HY in BxPc-3 and SU.86.86 cells and *ZEB1* by CM in MIA PaCa-2 and SU.86.86. None of the analyzed cell lines expressed the *TWIST1* and *ZEB2* genes. Surprisingly, EMT-TFs were frequently downregulated after 2-day treatment.

*CDH1* and *CDH2* genes were not expressed in the MIA PaCa-2 and PANC-1 cells. We confirmed the downregulation of several epithelial genes, mainly *OCLN* and *STEAP1,* in BxPc-3 and SU.86.86. The most significant upregulations of mesenchymal genes were found again in BxPC-3 and SU.86.86 cell lines, with the most significant changes in *FN1* and *ITGA5* due to HY and CM + HY exposure. *BMP2* was not expressed in MIA PaCa-2 and PANC-1, while its expression increased significantly due to HY and CM + HY in BxPC-3 and SU.86.86 cells. Gene expression changes in MIA PaCa-2 and PANC-1 cells were minor, mainly due to the relatively high number of silenced genes (*CDH1, STEAP1, CDH2, BMP2*, and *NID2*, while *DSC2* in MIA PaCa-2 only).

### 2.2. Global and Gene-Specific DNA Methylation in Individual Cell Lines

**Figure 4:**
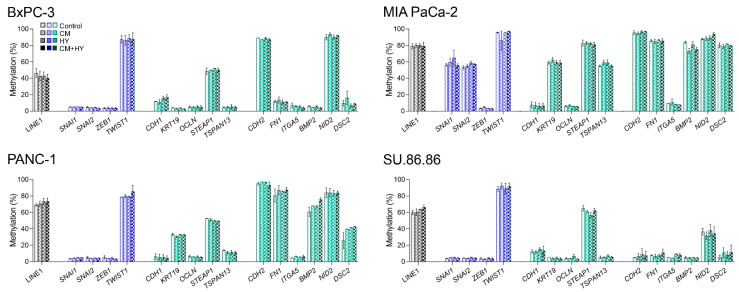
Global and promoter DNA methylation of individual genes and changes induced by 2-day exposure. EMT-TFs are highlighted by blue, EMT-related genes by green color; CM, conditioned media, HY, 1% hypoxia; no significant changes induced by a 2-day exposure to individual conditions were found.

The LINE-1, which represents a surrogate marker of global methylation level and promoter DNA methylation of 15 EMT genes, including TFs, was assessed in all studied cell lines using the quantitative pyrosequencing method described in detail in Materials and Methods (Figure 4). Global DNA methylation varied between 46% in BxPC-3, 79% in MIA PaCa-2, 69% in PANC-1, and 60% in SU.86.86 cells. Low DNA methylation was found for EMT-TFs, with methylation levels below 10%. However, *SNAI1* and *SNAI2* in MIA PaCa-2 cells and *TWIST1* in all cell lines were highly methylated (between 53% and 95%). Importantly, the DNA methylation level strongly correlated with gene expression in most studied genes. High promoter methylation was found particularly in silenced genes, in MIA PaCa-2 and PANC-1 cells, *TWIST1* (95%, 79%), *STEAP1* (83%, 53%), *CDH2* (95%, 95%), *BMP2* (84%, 61%), *NID2* (88%, 84%), respectively and *DSC2* (80% in MIA PaCa-2). Nevertheless, DNA methylation of the *CDH1* gene was low (between 6% and 12%) despite inhibited gene expression in MIA PaCa-2 and PANC-1 cells.

In BxPC-3, only four genes, *TWIST1* (87%), *CDH2* (89%), *NID2* (90%), and *STEAP1* (49%), were highly methylated, while in SU.86.86 cells, there were only three genes, *TWIST1* (89%), *NID2* (36%), and *STEAP1* (65%). Experimental conditions did not significantly affect global or gene-specific DNA methylation after a 2-day exposure.

### 2.3. Prolonged Treatment-Induced EMT-Related Gene Expression and DNA Methylation Changes

Due to negative findings for DNA methylation changes after 2-days, we extended exposure time up to 6 days to rule out the possibility that the exposure time was too short for the methylation changes to take effect. During this time, the cells survived in the given experimental conditions without subculturing and significantly reduced viability (by more than 20%).

**Figure 5:**
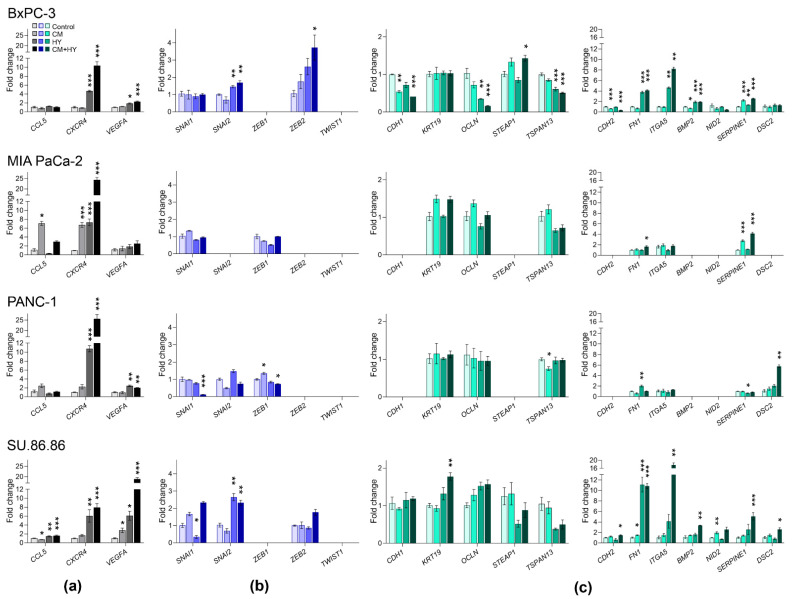
Changes in the expression of (**a**) inflammatory, (**b**) EMT-TFs, and (**c**) EMT-related genes after 6-day cell cultivation in inflammatory (CM), hypoxic (HY) conditions, and their combination (CM + HY), relative to Controls. Inflammatory genes are highlighted by grey, EMT-TFs by blue, and EMT-related genes by green color; CM, conditioned media, HY, 1% hypoxia; * *p* < 0.05, ** *p* < 0.01, *** *p* < 0.001.

Gene expression changes after 6-day exposure to experimental conditions (CM, HY and CM + HY) are provided in Figure 5 and Table S4. Interestingly, in BxPC-3 and MIA PaCa-2 cells after 6-days, the extent of *CXCR4* upregulation by HY and CM + HY was lower than after 2-days. On the other hand, *CXCR4* expression increased from 8.8-fold to 25.6-fold in PANC-1 and *VEGFA* from 5.8-fold to 18.9-fold in SU.86.86 by CM + HY (Figure 3a and Figure 5a). In addition, *CCL5* gene expression increased significantly due to CM in MIA PaCa-2 and all exposures in SU.86.86 cells. However, upregulation of *CCL5* was milder in comparison to other genes.

Individual cell lines exhibited considerable differences in the expression of EMT genes (Figure 5b,c). All genes except *ZEB1* and highly methylated *TWIST1* were expressed in BxPC-3 and SU.86.86 cells. However, many genes, including *CDH1*, were not expressed in MIA PaCa-2 and PANC-1 cells.

A significant decrease of three epithelial genes, *CDH1*, *OCLN*, and *TSPAN13*, was identified in BxPC-3 cells. On the other hand, expression of four mesenchymal genes, *FN1*, *ITGA5*, *BMP2*, and *SERPINE1*, increased significantly, while *CDH2* was downregulated. In MIA PaCa-2 cells, upregulation of more than two-fold was identified in the *SERPINE1* gene only. In PANC-1 cells, downregulation of *SNAI1* was accompanied by upregulation of *DSC2*. Although expression of epithelial genes in SU.86.86 cells did not change more than two-fold, most mesenchymal genes were upregulated mainly by CM + HY, namely *FN1*, *ITGA5*, *BMP2*, *SERPINE1*, and *DSC2*.

**Figure 6:**
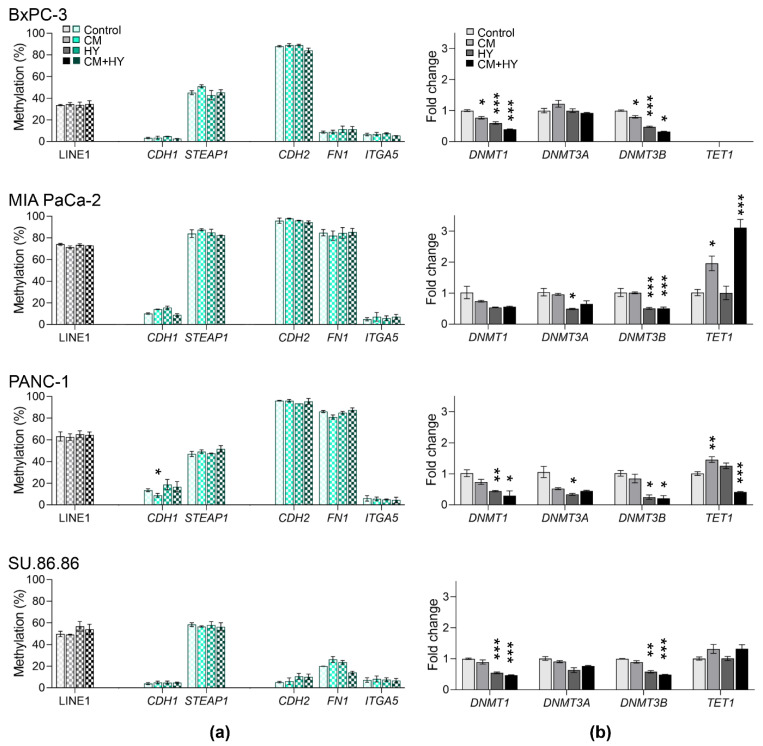
DNA methylation changes and gene expression after 6-day exposure; (**a**) global and gene-specific DNA methylation levels of selected genes; (**b**) expression of epigenetic effectors; CM, conditioned media, HY, 1% hypoxia; * *p* < 0.05, ** *p* < 0.01, *** *p* < 0.001.

Due to small DNA methylation changes after 2-day exposure, only LINE-1 and five representative genes with the most prominent expression changes were selected for analysis after 6-day exposure (Figure 6). Simultaneously, we assessed gene expression changes of three DNMTs and the *TET1* gene. *DNMT1* and *DNMT3B* decreased significantly in all cell lines except *DNMT1* in MIA PaCa-2. The *TET1* gene was not expressed in BxPC-3 cells; however, its expression increased significantly in MIA PaCa-2 by exposure to CM + HY while decreased by the same condition in PANC-1 cells. No changes were found in SU.86.86 cells.

**Figure 7:**
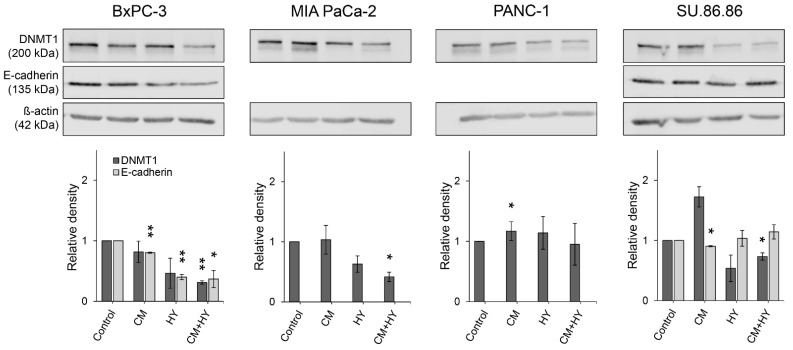
Western blot analysis and quantification by densitometry of DNMT1 and E-cadherin protein levels in the normoxic and hypoxic conditions enriched with conditioned media; CM, conditioned media, HY, 1% hypoxia; * *p* < 0.05, ** *p* < 0.01.

The expression of hallmark EMT protein, E-cadherin, and DNMT1, considered maintenance DNMT, were assessed in all cell lines by western blot. This method confirmed changes found by qRT-PCR (Figure 7), showing that E-cadherin was expressed in BxPC-3 and SU.86.86 cells only. Its expression decreased significantly after 6-day exposure in the BxPC-3 cell line and SU.86.86 by CM. DNMT1 expression decreased significantly by CM + HY in BxPC-3, MIA PaCa-2, and SU.86.86 cells, while it increased by CM in PANC-1.

### 2.4. Gene Expression and DNA Methylation Changes Induced by Decitabine

**Figure 8:**
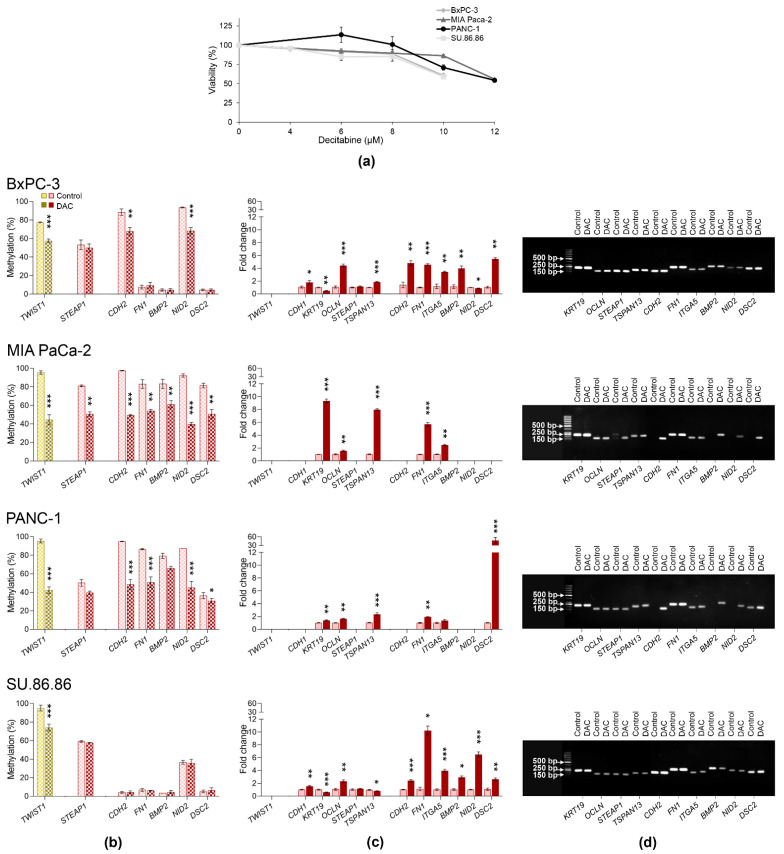
Changes in DNA methylation and gene expression induced by exposure to sub-cytotoxic decitabine (DAC) concentrations in PDAC cell lines. (**a**) Cell viability after DAC treatment; (**b**) DAC-induced DNA methylation changes in *TWIST1* (highlighted by yellow) and selected EMT-related genes (highlighted by red); (**c**) DAC-induced changes in the expression of *TWIST1* and EMT-related genes; (**d**) reactivation of gene expression; representative gels are shown for individual genes except for *TWIST1* and *CDH1*, * *p* < 0.05, ** *p* < 0.01, *** *p* < 0.001.

To confirm that the expression of studied genes was mediated by DNA methylation, we used DNMT inhibitor decitabine (DAC) (Figure 8, Table S5). Non-cytotoxic DAC concentrations (cell viability over 80%) were selected based on the results of the luminescence assay (Figure 8a). Given the mode of action and low stability, DAC was added daily for 3 days to allow cell division. Due to the expected decrease of DNA methylation by DAC, only seven highly methylated genes were analyzed for DAC-induced DNA methylation changes, and genes with low promoter methylation were excluded from pyrosequencing analysis (Figure 8b). However, *TWIST1* and all EMT-related genes were assessed for gene expression changes (Figure 8c). DAC efficiently decreased DNA methylation in the majority of highly methylated genes. A significant decrease was found for *TWIST1*, *CDH2*, and *NID2* in BxPC-3; for all studied genes in MIA PaCa-2; *TWIST1*, *CDH2*, *FN1*, *NID2*, and *DSC2* in PANC-1 cells, and *TWIST1* in SU.86.86.

Gene expression of all studied genes except for *STEAP1* increased significantly after DAC treatment in BxPC-3 cells, with more than two-fold increase in *OCLN*, *CDH2*, *FN1*, *ITGA5*, *BMP2*, and *DSC2*. In MIA PaCa-2 five genes were upregulated, *KRT19*, *TSPAN13*, *FN1*, and *ITGA5*. In PANC-1 it was *TSPAN13* and *DSC2*. Finally, in SU.86.86 cell line *OCLN*, *CDH2*, *FN1*, *ITGA5*, *BMP2*, *NID2*, and *DSC2* genes were upregulated.

Importantly, DAC reactivated gene expression of all silenced genes (Figure 8d), including *TWIST1* in all cell lines, *CDH1* in MIA PaCa-2, and PANC-1. Moreover, *STEAP1*, *CDH2*, *BMP2*, *NID2*, and *DSC2* were reactivated in the MIA PaCa-2 cell line, and *CDH2*, *STEAP1*, *BMP2*, in PANC-1 cells. However, significant upregulation was also found in the genes with low methylation levels, whose methylation did not change significantly, e.g., *FN1*, *BMP2*, and *DSC2* in BxPC-3 cells, and *CDH2, FN1, BMP2*, and *DSC2* in SU.86.86 cells.

**Figure 9:**
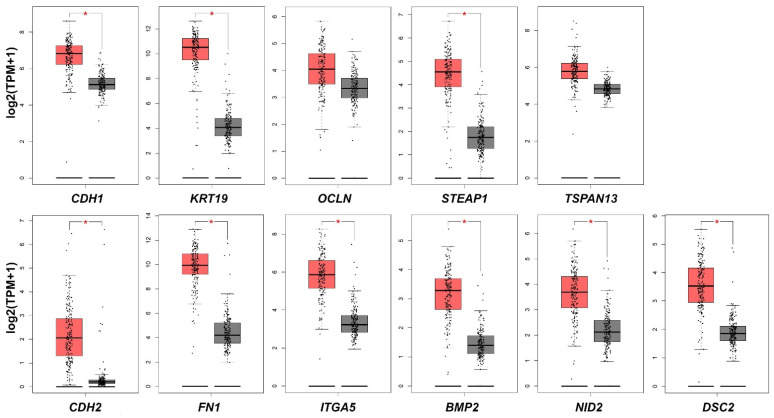
Comparison of studied EMT-related genes mRNA expression in PDAC and normal pancreatic tissues. * *p* < 0.05. The expression level is described as log2(TPM + 1). Red highlighted are PDAC tissues (n = 179), grey are normal pancreatic tissues (n = 171); TPM, transcript per million.

To evaluate the potential translational significance of our findings, we assessed the difference in the expression of analyzed EMT-related genes between PDAC and normal pancreatic tissues using The online Gene Expression Profiling Interactive Analysis (GEPIA) tool. GEPIA is a valuable and highly cited resource for gene expression analysis based on tumor and normal samples from The Cancer Genome Atlas (TCGA) and the Genotype-Tissue Expression (GTEx) databases (GEPIA (Gene Expression Profiling Interactive Analysis). Available online: http://gepia.cancer-pku.cn/ (accessed on 16 December 2021)) [33]. Based on the available data, mRNA expression of nearly all analyzed genes, except for *OCLN* and *TSPAN13,* was significantly upregulated in PDAC samples (Figure 9).

## 3. Discussion

Dense desmoplastic fibrotic stroma, the rapid proliferation of cancer cells, and poor vascularization contribute to the hypoxic microenvironment of PDAC [13]. Repression of E-cadherin and other genes involved in cell–cell and cell–basal membrane contacts are among the EMT features, leading to loss of epithelial characteristics, acquisition of a mesenchymal-like phenotype, and a worse prognosis. In addition to the EMT-TFs and cadherins, the role of other EMT-related genes has not been elucidated in PDAC.

In the present study, we focused on hypoxia- and inflammation-triggered gene expression changes of EMT genes and the role of DNA methylation in their regulation. Gene expression was modulated in a cell line-specific manner, with BxPC-3 cells manifesting the highest response to experimental conditions. BxPC-3 is the squamous or more basal-like human cell line, expressing the oncogenic ΔN form of TP63 (ΔNp63), present in human primary PDAC samples of this subtype. Interestingly, we found a high degree of similarity in gene expression and promoter DNA methylation patterns between BxPC-3 and metastatic SU.86.86 cells, the same as between MIA PaCa-2 and PANC-1 cells. Unique molecular features, including epithelial-mesenchymal phenotype and neuroendocrine differentiation attributed to MIA PaCa-2 and PANC-1 [34], together with divergent genetic profiles, may be responsible for a distinct response of the studied cell lines to experimental conditions [35].

Systemic and local chronic inflammation might enhance the risk of PDAC. The dynamic crosstalk between inflammatory and cancer cells is maintained by soluble mediators, cytokines, and chemokines, which are synthesized by the host tumor and stromal cells. They were shown to play an essential role in cellular proliferation, angiogenesis, metastasis, and immune evasion [36]. In agreement with the demonstrated role of hypoxia in inducing CXCR4 expression in cancer, we found *CXCR4* the most significantly upregulated gene by hypoxic conditions, independently of inflammatory stimuli. Accordingly, the expression of CXCR4 mediated the development of liver and lung metastasis in the pancreatic cancer animal model [37]. A positive correlation was documented between CXCR4 expression and PDAC progression, including hematogenous dissemination [38]. However, the relationship between the expression of CXCR4 in PDAC and clinicopathological parameters remains inconclusive. Although several studies described CXCR4 overexpression as a robust prognostic marker correlated with the risk of lymph node involvement and distant metastasis [39], recent findings from more than 3600 PDAC samples documented a higher CXCR4 expression in primary tumors than distant metastases [40]. CCL5 was one of 3 inflammatory cytokines deregulated in more than one cell line herein. The CCL5/ C-C motif chemokine receptor 5 (CCR5) axis gains increasing attention due to its involvement in tumor progression through multiple mechanisms, including immunosuppressive polarization, metabolic reprogramming, and ECM remodeling, facilitating migration and invasion of tumor cells [41]. The CCL5 has been identified as a key chemokine for Treg cells infiltration in PDAC. Moreover, besides elevated expression of CCL5 in poorly differentiated PDAC tissues compared to non-neoplastic and moderately differentiated, CCL5/CCR5 axis interaction was shown to promote migratory and invasiveness of PDAC BxPC-3, MIA PACa-2, and AsPC-1 cells [42]. Tumor proliferation is associated with the expression of pro-angiogenic factors, particularly VEGF. VEGF-A/VEGFR-2 signaling was shown to play a crucial role in the motility of pancreas cancer cells [43]. Accordingly, high expression of VEGFA was associated with a worse prognosis in PDAC [44].

Herein we found several EMT-related genes induced by hypoxia. This upregulation occurred only in cell lines and genes with low promoter DNA methylation. Although inflammation- and hypoxia-induced DNA methylation changes were negligible, differences in global and gene-specific DNA methylation between studied primary cell lines of the same origin (derived from pancreatic epithelial tissues) suggest an essential role of DNA methylation in PDAC tumorigenesis. However, in in vitro models, tumor cells acquire stable epigenetic marks after sustained cultivation of tumor cells under EMT-inducing conditions [45].

Furthermore, the regulatory function of DNA methylation in gene expression regulation was confirmed by the reactivation of silenced genes by DAC. This DNMT inhibitor is a deoxycytidine analog typically used to reactivate gene expression silenced by promoter methylation [46]. DAC incorporation into DNA leads to depletion of DNMT1 and passive demethylation. DAC induces gene expression changes also indirectly via demethylation of upstream genes, regulatory elements, or changes in histone modifications [47]. Although a significant decrease of DNA methylation accompanied reactivation of silenced genes, low DNA methylation levels of several upregulated genes, among them *CDH1*, suggest an indirect effect of DAC on their expression.

By default, in epithelial tissue-derived tumors, a reduction of epithelial genes and induction of mesenchymal phenotype is associated with EMT and higher tumor proliferation, motility, and metastasis. Among other reasons, high methylation levels of nearly all mesenchymal genes except for *ITGA5* in MIA PaCa-2 and PANC-1 cells can explain their resistance to experimental conditions. Herein, we discuss only genes differentially expressed in PDAC tissues compared to controls in TCGA dataset. Besides cadherins, whose role is well established in EMT and PDAC pathogenesis, we mainly focus on mesenchymal genes or those with a somewhat controversial role in EMT not associated yet with PDAC pathogenesis. The most upregulated *FN1* gene encodes the glycoprotein fibronectin found in the ECM, interacting with proteins such as collagen, fibrin, proteoglycans, and others [48]. Hypoxic conditions significantly increased *FN1* expression in BxPC-3 and SU.86.86 PDAC cell lines characterized by low promoter methylation. In agreement with previously published findings, FN1 induction by hypoxia directly correlated with the expression of its integrin receptor ITGA5 [49]. FN1 is primarily expressed in fibroblasts but can also be produced by other cell types, including cancer and endothelial cells [50]. Its expression is significantly increased in many solid tumors, including PDAC [51], promoting progression and metastasis [52]. High FN1 expression in PDAC tissues correlates with higher tumor weight, more advanced disease, and poorer prognosis after resection [53]. TGF-β stimulates FN1 expression and its transport to the extracellular space, where it participates in the EMT process. Knockdown of major TFs of the TGF-β pathway, SNAI1/2, and SMAD4, led to decreased FN1 expression, consequent EMT inhibition, and decreased tumor cell motility [54]. FN1 and ITGA5 also play an important role in tumor angiogenesis, although the mode of action has not been elucidated [55]. ITGA5 has been shown to potentiate the aggressiveness of cancer cells and their resistance to chemotherapy in animal models [56]. Increased methylation of the *ITGA5* gene has been associated with lower expression and increased invasiveness in breast tumors. However, its inactivation resulting in inhibition of cell division suggests diverse roles of ITGA5 [57]. The *STEAP1* belongs to the group of metalloreductases and is involved in tumor cell proliferation and suppresses apoptosis [58]. It is overexpressed in several types of human tumor tissues and cell lines, including tumors of the colon, pancreas, ovary, testis, and breast [59]. The role of this protein in cancer is controversial. While its expression inhibited metastasis in breast cancer, it was correlated with metastasis and EMT induction in lung adenocarcinoma [60,61]. In gastric tumors, the upregulation of *STEAP1* increased cell proliferation, migration, and invasion [62]. The *DSC2* gene is essential for desmosome formation in epithelial cells and is involved in epithelial morphogenesis, differentiation, wound healing, cell apoptosis, migration, and proliferation [63]. Low DSC2 expression has been reported to promote invasiveness and is involved in EMT in several types of epithelial tissue-derived tumors, including PDAC [64]. Highly differentiated PDAC tissues were also characterized by higher DSC2 expression compared to less differentiated ones, in which the complete absence of DSC2 was often observed [65]. The *NID2* gene encodes one of the basic components of the basement membrane and plays a key role in embryogenesis and the development of malignant tumors. In vitro experiments suggest that NID2 promotes invasiveness and migration in gastric carcinoma-derived tumor cells, where it was significantly overexpressed compared to healthy tissues [66]. Increased NID2 expression also correlated significantly with overall survival in gastric cancer patients [67]. Abnormal hypermethylation of the *NID2* promoter associated with suppression of its expression is known in aggressive types of breast tumors [68]. BMP2 is a growth factor that plays an important role in PDAC carcinogenesis [69]. It has been investigated as a potential prognostic marker in PDAC, but no significant correlation with survival or prognosis has been reported [70]. The BMP2 protein participates in the initiation and progression of several types of solid tumors, and its increased expression in the PDAC cell line PANC-1 has led to increased proliferation in both in vitro and in vivo models, presumably through impaired autocrine signaling [71]. BMP2 can also induce the EMT process and increase invasiveness in the PANC-1 cell line by activating the PI3K/Akt pathway [72]. The high level of BMP2 gene methylation in colorectal cancer is a negative prognostic marker typical for the third stage of the disease [73].

Hypoxia and inflammation play an essential role in the pathogenesis of PDAC, causing acidosis and the formation of reactive oxygen species and inducing genetic instability in pancreatic epithelial cells. Epigenomic landscapes explain the progression of PDAC into classical or more aggressive basal subtypes. Moreover, EMT plasticity suggests that the epigenetic landscapes are implicated in the dynamic events underlying mesenchymal and intermediate phenotypes responsible for tumor cell dissemination. This work shed light on the role of DNA methylation in the transcriptional regulation of several EMT genes. DNA methylation-mediated reactivation of silenced genes has a critical translational impact. However, further studies are warranted to investigate epigenetic drug efficacy in synergy with other anticancer therapies and possible off-target effects.

## 4. Materials and Methods

### 4.1. Pancreatic Cancer Cell Lines

In the present study, we used four epithelial PDAC cell lines, BxPC-3, MIA PaCa-2, PANC-1 derived from primary adenocarcinoma, and SU.86.86 derived from liver metastasis. The clinical course of the donor patients, site of derivation, histopathological appearance, and differentiation were described elsewhere [35]. These cells harbor different genetic backgrounds reflected in their phenotypic characteristics. BxPC-3 cells are KRAS negative, while MIA PaCa-2 possess G12C, PANC-1 G12D, and SU.86.86 G12D KRAS mutation, all have a homozygous deletion in exons 2 and 6 of p16 and variable mutations of *TP53*. Except for BxPC-3 with a homozygous deletion in exons 1–11, they do not carry *SMAD4* mutations. All cells were cultivated at 37 °C in a humidified atmosphere (5 % CO_2_) and maintained in high-glucose (4.5 g/L) Dulbecco’s modified Eagle medium (DMEM, PAA Laboratories GmbH, Pasching, Austria) supplemented with 10% fetal bovine serum (FBS, Biochrom AG, Berlin, Germany), 2 mM glutamine (PAA Laboratories GmbH), and 10 μg/mL gentamicin (Sandoz, Nürnberg, Germany). The cell cultures were regularly tested for mycoplasma contamination by PCR.

### 4.2. Cell Viability

For viability assay, the cells were seeded into 96-well plates at a density of 5.5 × 10^3^ cells/well for BxPC-3, 1.9 × 10^3^ cells/well for MIA PaCa-2, 3.0 × 10^3^ cells/well for PANC-1, 4.8 × 10^3^ cells/well for SU.86.86 and exposed to different concentrations of DAC (MedChem Express, Shanghai, China) (4–12 µM) added every 24 h in a total of 72 h. To assess the relative viability of cells, CellTiter-Glo^®^ Luminescent Cell Viability Assay (Promega Corporation, Madison, WI, USA) and GloMax^®^ Discover Microplate Reader (Promega Corporation, Madison, WI, USA) were used. Cell viability was determined as the luminescence intensity relative to untreated control cells (set to 100%). The results are presented as means ± SEM from at least two independent experiments in quadruplicates.

### 4.3. Cell Exposure

The pancreatic cells were indirectly co-cultured with contact-activated stromal fibroblasts to establish an experimental fibro-inflammatory in vitro model [74]. As previously described, activated fibroblasts were characterized by the production of inflammation-associated cytokines and growth factors, e.g., IL-1, IL-6, IL-8, IL-11, LIF, GM-CSF, and COX-2 related- prostaglandins [32]. All PDAC cell lines were cultivated with or without a conditioned medium (CM) for 2 and 6 days in normoxic or hypoxic conditions. Hypoxic experiments were carried out in the hypoxic workstation (Ruskinn Technologies, Bridgend, UK) in a 1% O_2_, 2% H_2_, 5% CO_2_, 92% N_2_ atmosphere at 37 °C.

For detection of the DAC-induced (DAC, MedChem Express, Shanghai, China ) gene expression and DNA methylation changes, the cells were seeded on Petri dishes (60 mm) at a 250 × 10^3^ cells/dish density. Subsequently, subcytotoxic concentrations of DAC (cell viability around 80%) were added every 24 h in a total of 72 h (for BxPC-3 and SU.86.86 6 µM DAC; for MIA PaCa-2 and PANC-1 8 µM DAC).

### 4.4. Expression Arrays

After exposure to individual experimental conditions, RNA was extracted from cell pellets using miRNeasy Mini Kit (Qiagen, Hilden, Germany). The RNA quality and quantity were assessed using NanoDrop^®^ ND-1000 spectrophotometer (Thermo Fisher Scientific, Wilmington, DE, USA), and 2.5 µg of total RNA was reverse transcribed by RT² First Strand Kit (Qiagen, Hilden, Germany), following manufacturer instructions.

Cancer Inflammation and Immunity Crosstalk RT^2^ Profiler PCR Array (PAHS-181Z; SABiosciences, Frederick, MD, USA) was used to analyze 84 genes or biological pathways involved in mediating communication between tumor cells and the cellular mediators of inflammation and immunity. In addition, 84 genes involved in tumor metastasis or stem cell differentiation and development were analyzed by Human Epithelial to Mesenchymal Transition RT² Profiler PCR Array (PAHS-090ZA; SABiosciences, Frederick, MD, USA). According to the manufacturer’s protocol, real-time PCR was performed using RT^2^ Profiler PCR Arrays containing pre-designed primer sets in combination with RT^2^ SYBR Green/ROX PCR Master Mix (Qiagen, Hilden, Germany). PCR reaction was performed on Bio-Rad CFX96 real-time PCR detection system (Bio-Rad, Hercules, CA, USA) using a 3 step cycling program: 95 °C for 10 min, 45 cycles at 95 °C for 15 s and 60 °C for 60 s. Data analysis was performed using web-based RT^2^ Profiler PCR Array Data Analysis version 3.5 (Gene globe data analysis. Available online: https://geneglobe.qiagen.com/us/analyze (accessed on 16 December 2021)). The expression levels of target genes were normalized relative to the values obtained for housekeepers (*ACTB, B2M, GAPDH, HPRT1*, and *RPLP0*) and quantified against controls. At least a two-fold change identified in two or more cell lines was considered for validation. To represent fold-change results in a biologically meaningful way, fold regulation values were calculated for FC below 1, as −1/fold change.

### 4.5. qRT-PCR Analysis

Total RNA from PDAC cell lines under individual culture conditions was isolated using the NucleoSpin^®^ RNA kit (Machery-Nagel, Düren, Germany). The RNA quality and quantity were measured using NanoDrop^®^ ND-1000 spectrophotometer (Thermo Fisher Scientific, Wilmington, DE, USA). Revert Aid TM H minus first-strand cDNA synthesis kit (Thermo Fisher Scientific, Loughborough, UK) was used for reverse transcription of total RNA (up to 4 µg from each sample).

qRT-PCR analyses were performed with TaqMan^®^ assays (Thermo Fisher Scientific Loughborough, UK) or individually designed gene primers (Table S1). The following TaqMan gene expression assays, *CDH1* (Hs01023894_m1), *TWIST1* (Hs00361186_m1), *SNAI1* (Hs00195591_m1), *SNAI2* (Hs00161904_m1), *ZEB1* (Hs01566408_m1), *DNMT1* (Hs00945875_m1), *DNMT3A* (Hs01027166_m1), *DNMT3B* (Hs00171876_m1) and *TET1* (Hs00286756_m1) were employed, including *HPRT1* (Hs02800695_m1) used for normalization. The reaction mix contained 10 µL of 2× Taq-Man gene expression master mix (Thermo Fisher Scientific, Loughborough, UK), 1 µL of 20× TaqMan^®^ Assay, 9 µL of 50 ng cDNA template, and ultrapure DNase/RNase-free water. Amplification was performed on a Bio-Rad CFX96 real-time PCR detection system (Bio-Rad, Hercules, CA, USA) using the cycling program: 50 °C for 2 min, 95 °C for 10 min and 40 cycles at 95 °C for 15 s followed by 56–63 °C for 60 s, depending on primers (amplification temperatures for individual primer pairs are listed in Table S1). All samples were analyzed in triplicates. qRT-PCR analysis of *ZEB2*, *FN1*, *SERPINE1*, *CDH2*, *KRT19*, *STEAP1*, *OCLN*, *DSC2*, *NID2*, *TSPAN13*, *BMP2*, *ITGA5*, *VEGFA*, *EGF*, *CXCR4*, and *CCL5* genes was performed with individually designed primers, using *HPRT1* for normalization (Table S1). The reaction mixture consisted of 7.5 µL of 2× GoTaq^®^ qPCR Master Mix (Promega, Madison, WI, USA), 1 µL (0.67 μM) forward primer, 1 µL (0.67 μM) reverse primer, 4.5 µL ultrapure DNase/RNase-free water, and 50 ng cDNA. Amplification was carried out on a Bio-Rad CFX96 real-time PCR detection system (Bio-Rad, Hercules, CA, USA), all samples were analyzed in triplicates. Samples with ct values over 35 were considered unexpressed. Relative mRNA expression was calculated using the 2^−ΔΔCt^ method [75]. Statistical analysis was applied to the dCt values. FCs above 2.0 and below 0.5 with *p* < 0.05 were discussed only.

### 4.6. Western Blot

Samples from all studied cell lines exposed to experimental conditions for 6 days were used for protein isolation and Western blot analysis. For protein isolation, cells were lysed with RIPA buffer (Cell Signaling Technology, Danvers, MA, USA) supplemented with PhosSTOP™ (Roche, (Mannheim, Germany) and Complete™ Protease Inhibitor Cocktail (Roche, (Mannheim, Germany). Total protein concentration was determined by Pierce™ BCA Protein Assay Kit (Thermo Fisher Scientific, Waltham, MA, USA). A total amount of 40 µg of proteins per sample was used for analysis. Samples were diluted in 4× Laemmli Sample Buffer (Bio-Rad, Hercules, CA, USA) prior to the use and denatured by heating at 95 °C for 5 min. Proteins were separated by SDS-PAGE (7.5–10%) and transferred to nitrocellulose membrane (Whatman, Dassel, Germany). The membranes were blocked in 5% non-fat milk diluted in TBS (20 mM Tris, 150 mM NaCl) for 1 h and incubated overnight at 4 °C with primary antibodies against E-cadherin (Cell Signaling Technology, Danvers, MA, USA, cat. No. 14472) and DNMT1 (Cell Signaling Technology, Danvers, MA, USA, cat. No. 5032) diluted 1:1000 in 5% non-fat milk in TBST (20 mM Tris, 150 mM NaCl, 0.1 % Tween 20). Membranes were incubated with a primary antibody against β-actin (Sigma Aldrich, Taufkirchen, Germany, cat.no. A1978) diluted 1:4000 in 5% non-fat milk in TBST for 1 h at room temperature. Therefore, they were incubated with Goat anti-Rabbit IgG (H + L) Highly Cross-Adsorbed Secondary Antibody, Alexa Fluor 680 or Goat anti-Mouse IgG (H + L) Highly Cross-Adsorbed Secondary Antibody, Alexa Fluor 680 (Thermo Fisher Scientific, Waltham, MA, USA) secondary antibodies diluted 1:10,000 in 5% non-fat milk in TBST for 1 h at room temperature. Proteins were visualized by Odyssey^®^ Fc (LI-COR Biosciences, Lincoln, NE, USA) imaging system, and densitometry was performed using ImageJ/Fiji software. The results represent the ratio of protein to loading control (B-actin) relative to the control sample of two independent experiments.

### 4.7. DNA Methylation Analysis

Genomic DNA from studied PDAC cell lines (1–2 × 10^6^ cultured cells) was isolated using a FlexiGene DNA Kit (Qiagen, Hilden, Germany). DNA concentration and purity were measured using NanoDrop^®^ ND-1000 spectrophotometer (Thermo Scientific, Wilmington, DE, USA). For sodium bisulfite treatment of extracted DNA (2 µg), EpiTect Bisulfite kit (Qiagen, Hilden, Germany) was used, following the provided protocol. EpiTect Bisulfite kit enables complete conversion of unmethylated cytosines to uracils, while methylated cytosines remain unaffected.

DNA methylation profiles of 15 top-ranked genes (*SNAI1*, *SNAI2*, *TWIST1*, *ZEB1*, *CDH1*, *FN1*, *CDH2*, *KRT19*, *STEAP1*, *OCLN*, *DSC2*, *NID2*, *TSPAN13*, *BMP2*, *ITGA5*) and methylation level of the long-interspersed nucleotide element 1 (LINE-1) were evaluated by the quantitative pyrosequencing method, carried out on a PyroMark Q24 platform, using PyroMark Gold Q24 Reagents (Qiagen GmbH, Hilden, Germany). Pyrosequencing assays for all genes were designed using the PyroMark assay design software (Qiagen GmbH, Hilden, Germany), primer sequences and PCR conditions are listed in Table S2. Designed assays were validated following the manufacturer’s instructions. Methylation analyses were repeated twice. Between 2 and 7, CpGs were analyzed in each gene in the CpG islands of the promoter regions flanking the transcription start site. The results are presented as the percentage of average methylation in all CpG sites in each gene.

Global DNA methylation was analyzed with the PyroMark Q24 CpG LINE-1 kit (Qiagen, Hilden, Germany), allowing quantification of the methylation levels of three CpG sites in positions 331 to 318 of the LINE-1 sequence (GenBank accession number X58075). The PCR reactions were performed by the PyroMark PCR Kit (Qiagen, Hilden, Germany) following the manufacturer’s instructions. Data analysis was performed by PyroMark Q24 2.0.6. software (Qiagen, Hilden, Germany).

### 4.8. Validation of mRNA Expression of Studied Genes between PDAC and Normal Tissue

The online tool GEPIA. Available online: http://gepia.cancer-pku.cn/ (accessed on 16 December 2021) [76] was used to validate the mRNA expression levels of the screened genes between PDAC and normal pancreatic tissues.

### 4.9. Statistical Analysis

Normality of distribution was tested by the Shapiro–Wilk test. Significant differences between normally distributed data were assessed by Student *t*-test or one-way analysis of variance (ANOVA) and Bonferroni or Tamhane post-hoc tests depending on assumed variances. Non-normally distributed data were evaluated using Mann–Whitney U-test or Kruskal–Wallis test followed by Dunn of Dunn–Bonferroni post-hoc methods. Data were analyzed using the SPSS software package version 23 (IBM SPSS, Inc., Chicago, IL, USA). Differences with *p* < 0.05 were considered statistically significant.

## Data Availability

All data supporting the reported results can be found as Supplementary Files.

## Supplementary Materials

The following supporting information can be downloaded at: https://www.mdpi.com/article/10.3390/ijms23042117/s1.
