# Supplementary material for: DNA Methylation Mediates EMT Gene Expression in Human Pancreatic Ductal Adenocarcinoma Cell Lines
Source: Int J Mol Sci. 2022 Feb 14;23(4):2117. doi: 10.3390/ijms23042117 (PMC8879087; doi:10.3390/ijms23042117)
Supplement: Supplementary file 1 [file ijms-23-02117-s001.zip › ijms-1561712-supplementary.pdf]

**Table S1**. qRT-PCR primers

| Gene | Orientation | Primer sequence (5 '→ 3') | Annelation temperature | Product length |
|---|---|---|---|---|
| *HPRT1* | F | GGACTAATTATGGACAGGACT | 60°C | 194 bp |
| | R | GCTCTTCAGTCTGATAAAATCTAC | | |
| *VEGFA* | F | ATCACGAAGTGGTGAAGTTC | 60°C | 265 bp |
| | R | TGCTGTAGGAAGCTCATCTC | | |
| *CXCR4* | F | GGGAACTTCCTATGCAAGGCAGTC | 59°C | 174 bp |
| | R | GATCCAGACGCCAACATAGACCAC | | |
| *CCL5* | F | CCTCGCTGTCATCCTCATT | 54°C | 97 bp |
| | R | GGCAATGTAGGCAAAGCA | | |
| *ZEB2* | F | TTTCCTGGCCCCATCCCTTT | 60°C | 184 bp |
| | R | GGTTGATGGGGCTTGTCATTCCT | | |
| *KRT19* | F | CCGAACCAAGTTTGAGACGG | 60°C | 221 bp |
| | R | AATCCACCTCCACACTGACC | | |
| *OCLN* | F | ACTCGAAGAAAGATGGACAGGT | 63°C | 152 bp |
| | R | TCCACATAGTCAGATGGGGGT | | |
| *STEAP1* | F | TGTCTCTGGGAATTGTGGGA | 60°C | 151 bp |
| | R | AATGCGTGTATTGTGCCCAG | | |
| *TSPAN13* | F | TTCGCGTGTTCCAAGAACTG | 63°C | 187 bp |
| | R | CAGCTCCAATCAGACCCACT | | |
| *CDH2* | F | GTCAGTGAAGGAGTCAGCAG | 60°C | 150 bp |
| | R | AGCTCTTGAGGAAAAGGTCC | | |
| *FN1* | F | ACCAACCTACGGATGACTCG | 60°C | 230 bp |
| | R | GCTCATCATCTGGCCATTTT | | |
| *ITGA5* | F | TGCCCCAAAAAGAGCGTCAG | 63°C | 172 bp |
| | R | TATGGGAGGGAGCGTTTGAAG | | |
| *BMP2* | F | ACATGCTAGACCTGTATCGCA | 63°C | 235 bp |
| | R | TGTTCTCGGAAAACCTGAAGC | | |
| *NID2* | F | TGTACCGAGAGGACACCTCC | 63°C | 197 bp |
| | R | AGATGCCAAAACTGCCTGGA | | |
| *SERPINE1* | F | AGCAACTTGCTTGGGAAAGGAGC | 60°C | 205 bp |
| | R | CGGGCGTGGTGAACTCAGTATAGTT | | |
| *DSC2* | F | TGTTTACGCTGGTCTGTGGG | 63°C | 178 bp |
| | R | CCACAAACTCCCTGAGCAGA | | |

**Table S2.** Pyrosequencing primers

| Gene | Orientation | Primer sequence (5 '→ 3') | Annelation | Product length | CpG sites |
|---|---|---|---|---|---|
| *SNAI1* | F | GTATTTGTTAGGGGAGTGGT | 56°C | 72 bp | 6 |
| | R | [Btn]-ACCACCCCCCTTTATCAC | | | |
| | seq. | GTTAGGGGAGTGGTTT | | | |
| *SNAI2* | F | [Btn]-GTTGGTTTGGTGTGGTGTAG | 56°C | 100 bp | 5 |
| | R | CCCTACCCCCCTAACTTCCAAATATAAT | | | |
| | seq. | CCTAACTTCCAAATATAATACAAC | | | |
| *ZEB1* | F | [Btn]-GTTGAAGTAGGATAATGGGGTTTGGA | 56°C | 150 bp | 8 |
| | R | TTCCCTTAACACAACAACACAAATCAAT | | | |
| | seq. | AGGAGGGTGGGGGAG | | | |
| *TWIST1* | F | GAAGGGGAGGGAAGGGG | 56°C | 97 bp | 3 |
| | R | [Btn]-TAACAATTCCTCCTCCCA | | | |
| | seq. | GGGAGGGAAGGGGGAG | | | |
| *CDH1* | F | ATTTTAGTAATTTTAGGTTAGAGGGTTA | 52°C | 106 bp | 7 |
| | R | [Btn]-ACCACAACCAATCAACAAC | | | |
| | seq. | ACCACAACCAATCAACAAC | | | |
| *KRT19* | F | ATTGGAGATAGTTGTAGGAAGTTATG | 56°C | 70 bp | 4 |
| | R | [Btn]-AATCCCAACTTCTAAAACCAAAATTACTC | | | |
| | seq. | AGTTGTAGGAAGTTATGG | | | |
| *OCLN* | F | [Btn]-TAGGGTGTGGGAAGTAGGAT | 56°C | 75 bp | 4 |
| | R | CCTCCTCCCTCCATAACCAATTCACCTAAA | | | |
| | seq. | ACCAATTCACCTAAAACTC | | | |
| *STEAP1* | F | AGAGTGGGTGAGTTTTTTGAAT | 56°C | 106 bp | 2 |
| | R | [Btn]-CTACACCCCTCCCAAAACTA | | | |
| | seq. | GTGTTTTTGTAAGGTTGG | | | |
| *TSPAN13* | F | GGGTTAGGTTTTAAAGGTAAGGATAAAG | 56°C | 103 bp | 5 |
| | R | [Btn] CCCCCCCAAACCATCTTAAAACCTATAATT | | | |
| | seq. | AGTAGTTGTTAGGGAATT | | | |
| *CDH2* | F | TAGATTGGGATAAAGATAATAGGATAGTAG | 56 °C | 95 bp | 3 |
| | R | [Btn]-CTTAATTACCAAACTTACCTCCACCTTACC | | | |
| | seq. | GGATAGTAGAGTTTTTGAG | | | |
| *FN1* | F | AGGGTTTTATTTTTGAGGTAGTTT | 56 °C | 93 bp | 4 |
| | R | [Btn]-ACAAATAATTCAACCCCAATCC | | | |
| | seq. | TGAGGTAGTTTGTTTTAGT | | | |
| *ITGA5* | F | TTGGGTTTTTGGAATTTTGAGT | 56°C | 106 bp | 4 |
| | R | [Btn]-CCAAACAACCCACTTCCAACTAAAACT | | | |
| | seq. | GGTTTTTGGAATTTTGAGTT | | | |
| *BMP2* | F | GGGGAAATAAGAGGTTAGATTTTGAT | 56°C | 84 bp | 6 |
| | R | [Btn]-CCTCCACTCCCTACTCTCA | | | |
| | seq. | GATTTTGTATTTGTTTTGGAT | | | |
| *NID2* | F | GTTTGGGGAGTAGGATGAGGTA | 56°C | 80 bp | 4 |
| | R | [Btn]-CAAAAAATCAAAATCTTCCCATCCC | | | |
| | seq. | GGAGTAGGATGAGGTAT | | | |
| *DSC2* | F | [Btn]-AGGGTTAGTAGGAGTAGT | 56°C | 81 bp | 4 |
| | R | CCTACCCCCAACCCTCTCCATA | | | |
| | seq. | ACCCTCTCCATAAAAAC | | | |

**Table S3.** Gene expression changes after 2 days of exposure

| Condition | | CM | | | HY | | | CM+HY | | |
|---|---|---|---|---|---|---|---|---|---|---|
| **Cell line** | **Gene** | **FC** | **SEM** | **p-value** | **FC** | **SEM** | **p-value** | **FC** | **SEM** | **p-value** |
| **BxPC-3** | *CCL5* | 0.74 | 0.03 | 1.000 | 0.54 | 0.02 | 0.247 | 0.45 | 0.00 | 0.013 |
| | *CXCR4* | 2.20 | 0.21 | 1.000 | 118.50 | 2.87 | <0.001 | 162.71 | 10.40 | <0.001 |
| | *VEGFA* | 1.76 | 0.09 | 0.002 | 5.41 | 0.86 | 0.083 | 4.38 | 0.27 | <0.001 |
| | *SNAI1* | 1.60 | 0.14 | 0.006 | 0.70 | 0.17 | 0.766 | 2.44 | 0.02 | <0.001 |
| | *SNAI2* | 2.05 | 0.11 | <0.001 | 1.19 | 0.27 | 0.993 | 0.70 | 0.02 | <0.001 |
| | *ZEB1* | 1.54 | 0.10 | 0.186 | 0.51 | 0.00 | 0.003 | 1.68 | 0.02 | 0.006 |
| | *ZEB2* | # | | | # | | | # | | |
| | *TWIST1* | # | | | # | | | # | | |
| | *CDH1* | 1.45 | 0.08 | 0.273 | 0.46 | 0.22 | 0.131 | 0.70 | 0.08 | 0.735 |
| | *KRT19* | 1.13 | 0.13 | 0.944 | 2.35 | 0.14 | 0.003 | 1.50 | 0.07 | 0.003 |
| | *OCLN* | 1.05 | 0.09 | 1.000 | 0.48 | 0.03 | <0.001 | 0.41 | 0.03 | <0.001 |
| | *STEAP1* | 1.17 | 0.07 | 0.349 | 0.54 | 0.04 | <0.001 | 0.69 | 0.11 | 0.232 |
| | *TSPAN13* | 0.85 | 0.02 | 0.002 | 1.08 | 0.03 | 0.365 | 1.03 | 0.15 | 1.000 |
| | *CDH2* | 0.60 | 0.03 | <0.001 | 1.51 | 0.05 | <0.001 | 0.72 | 0.06 | 0.002 |
| | *FN1* | 0.72 | 0.03 | 0.003 | 7.20 | 0.20 | <0.001 | 4.56 | 0.34 | 0.009 |
| | *ITGA5* | 1.53 | 0.05 | 1.000 | 9.80 | 0.32 | <0.001 | 11.55 | 0.91 | <0.001 |
| | *BMP2* | 0.87 | 0.06 | 0.406 | 2.86 | 0.28 | 0.016 | 2.69 | 0.05 | <0.001 |
| | *NID2* | 1.53 | 0.33 | 0.762 | 1.23 | 0.03 | 0.288 | 0.69 | 0.05 | 0.042 |
| | *SERPINE1* | 2.55 | 0.21 | 0.011 | 3.11 | 0.09 | 0.009 | 5.21 | 0.53 | 0.025 |
| | *DSC2* | 1.13 | 0.04 | 1.000 | 2.56 | 0.37 | 0.007 | 3.90 | 0.52 | <0.001 |
| **MIA PaCa-2** | *CCL5* | 3.96 | 0.21 | <0.001 | 2.35 | 0.40 | 0.155 | 2.15 | 0.06 | 0.002 |
| | *CXCR4* | 1.43 | 0.36 | 1.000 | 38.3 | 1.62 | <0.001 | 20.69 | 0.83 | <0.001 |
| | *VEGFA* | 2.58 | 0.54 | 0.236 | 1.05 | 0.23 | 1.000 | 1.21 | 0.24 | 0.967 |
| | *SNAI1* | 0.26 | 0.05 | <0.001 | 0.11 | 0.01 | <0.001 | 0.51 | 0.16 | 0.232 |
| | *SNAI2* | # | | | # | | | # | | |
| | *ZEB1* | 3.34 | 0.00 | <0.001 | 0.39 | 0.04 | 0.001 | 0.67 | 0.10 | 0.038 |
| | *ZEB2* | # | | | # | | | # | | |
| | *TWIST1* | # | | | # | | | # | | |
| | *CDH1* | # | | | # | | | # | | |
| | *KRT19* | 1.38 | 0.11 | 0.151 | 1.33 | 0.13 | 0.327 | 1.64 | 0.13 | 0.004 |
| | *OCLN* | 3.57 | 0.21 | 0.004 | 1.16 | 0.05 | 0.282 | 0.09 | 0.00 | <0.001 |
| | *STEAP1* | # | | | # | | | # | | |
| | *TSPAN13* | 0.58 | 0.04 | 0.003 | 1.08 | 0.05 | 1.000 | 0.40 | 0.14 | <0.001 |
| | *CDH2* | # | | | # | | | # | | |
| | *FN1* | 1.17 | 0.23 | 0.986 | 1.67 | 0.09 | 0.004 | 1.89 | 0.18 | 0.035 |
| | *ITGA5* | 0.95 | 0.03 | 1.000 | 0.46 | 0.02 | <0.001 | 0.36 | 0.14 | <0.001 |
| | *BMP2* | # | | | # | | | # | | |
| | *NID2* | # | | | # | | | # | | |
| | *SERPINE1* | 10.39 | 0.37 | <0.001 | 0.78 | 0.06 | 0.061 | 1.84 | 0.16 | 0.016 |
| | *DSC2* | # | | | # | | | # | | |
| **PANC-1** | *CCL5* | 1.60 | 0.15 | 0.008 | 1.19 | 0.07 | 1.000 | 1.21 | 0.11 | 1.000 |
| | *CXCR4* | 0.66 | 0.22 | 1.000 | 12.78 | 0.98 | <0.001 | 8.88 | 1.34 | <0.001 |
| | *VEGFA* | 1.04 | 0.11 | 1.000 | 1.04 | 0.07 | 0.997 | 1.02 | 0.05 | 1.000 |
| | *SNAI1* | 0.81 | 0.01 | 0.020 | 0.49 | 0.15 | 0.155 | 0.40 | 0.12 | 0.067 |
| | *SNAI2* | 1.55 | 0.06 | <0.001 | 1.07 | 0.05 | 0.926 | 0.78 | 0.13 | 0.598 |
| | *ZEB1* | 1.58 | 0.37 | 0.772 | 0.94 | 0.11 | 0.998 | 0.99 | 0.07 | 1.000 |
| | *ZEB2* | # | | | # | | | # | | |

| | | | | | | | | | | |
|---|---|---|---|---|---|---|---|---|---|---|
| | *TWIST1* | # | | | # | | | # | | |
| | *CDH1* | # | | | # | | | # | | |
| | *KRT19* | 0.92 | 0.12 | 0.990 | 0.74 | 0.05 | 0.008 | 0.67 | 0.03 | <0.001 |
| | *OCLN* | 1.85 | 0.26 | 0.002 | 0.96 | 0.07 | 1.000 | 0.86 | 0.04 | 1.000 |
| | *STEAP1* | # | | | # | | | # | | |
| | *TSPAN13* | 0.83 | 0.07 | 0.340 | 1.17 | 0.17 | 0.939 | 0.96 | 0.06 | 0.991 |
| | *CDH2* | # | | | # | | | # | | |
| | *FN1* | 1.14 | 0.10 | 1.000 | 1.20 | 0.10 | 1.000 | 1.00 | 0.07 | 1.000 |
| | *ITGA5* | 1.03 | 0.12 | 1.000 | 1.01 | 0.15 | 1.000 | 0.95 | 0.13 | 0.999 |
| | *BMP2* | # | | | # | | | # | | |
| | *NID2* | # | | | # | | | # | | |
| | *SERPINE1* | 1.79 | 0.34 | 0.040 | 1.58 | 0.12 | 0.228 | 1.53 | 0.05 | 0.337 |
| | *DSC2* | 1.50 | 0.27 | 0.696 | 5.60 | 0.47 | <0.001 | 4.04 | 0.19 | <0.001 |
| **SU.86.86** | *CCL5* | 1.43 | 0.52 | 0.008 | 3.13 | 0.49 | <0.001 | 3.08 | 0.85 | 0.003 |
| | *CXCR4* | 1.00 | 0.19 | 1.000 | 5.56 | 0.24 | <0.001 | 7.22 | 0.61 | <0.001 |
| | *VEGFA* | 8.13 | 0.06 | <0.001 | 3.82 | 0.36 | 0.003 | 5.80 | 0.25 | <0.001 |
| | *SNAI1* | 1.18 | 0.09 | 1.000 | 1.49 | 0.23 | 0.149 | 2.93 | 0.12 | <0.001 |
| | *SNAI2* | 1.64 | 0.17 | 0.001 | 0.40 | 0.10 | 0.001 | 0.42 | 0.02 | 0.002 |
| | *ZEB1* | 2.13 | 0.14 | <0.001 | 0.59 | 0.10 | 0.019 | 0.67 | 0.02 | <0.001 |
| | *ZEB2* | # | | | # | | | # | | |
| | *TWIST1* | # | | | # | | | # | | |
| | *CDH1* | 1.05 | 0.08 | 1.000 | 2.52 | 0.14 | <0.001 | 1.32 | 0.41 | 1.000 |
| | *KRT19* | 1.39 | 0.28 | 0.840 | 0.78 | 0.18 | 0.867 | 0.89 | 0.14 | 0.979 |
| | *OCLN* | 1.46 | 0.11 | 0.070 | 0.50 | 0.04 | <0.001 | 0.95 | 0.22 | 1.000 |
| | *STEAP1* | 1.68 | 0.17 | <0.001 | 0.36 | 0.10 | <0.001 | 0.38 | 0.08 | <0.001 |
| | *TSPAN13* | 1.12 | 0.11 | 1.000 | 0.74 | 0.14 | 0.694 | 0.74 | 0.16 | 0.851 |
| | *CDH2* | 2.04 | 0.22 | 0.055 | 1.05 | 0.04 | 0.864 | 1.43 | 0.13 | 0.151 |
| | *FN1* | 1.81 | 0.14 | 0.009 | 8.73 | 0.26 | <0.001 | 19.14 | 1.21 | <0.001 |
| | *ITGA5* | 1.66 | 0.04 | 1.000 | 8.37 | 0.30 | <0.001 | 13.34 | 0.61 | <0.001 |
| | *BMP2* | 1.25 | 0.12 | 1.000 | 7.09 | 0.51 | <0.001 | 9.13 | 0.37 | <0.001 |
| | *NID2* | 1.17 | 0.13 | 0.829 | 0.43 | 0.06 | <0.001 | 1.30 | 0.30 | 0.948 |
| | *SERPINE1* | 3.01 | 0.35 | 0.054 | 4.89 | 0.75 | <0.001 | 5.80 | 0.34 | <0.001 |
| | *DSC2* | 1.42 | 0.07 | <0.001 | 0.80 | 0.04 | 0.065 | 1.11 | 0.08 | 1.000 |

# Unexpressed in Controls (DMEM under normoxic conditions); CM, conditioned media in normoxia; HY, DMEM in hypoxia; CM+HY, CM in hypoxia

**Table S4.** Gene expression changes after 6 days of exposure

| Condition | | CM | | | HY | | | CM+HY | | |
|---|---|---|---|---|---|---|---|---|---|---|
| **Cell line** | **Gene** | **FC** | **SEM** | **p-value** | **FC** | **SEM** | **p-value** | **FC** | **SEM** | **p-value** |
| **BxPC-3** | *CCL5* | 0.81 | 0.19 | 0.825 | 1.26 | 0.08 | 0.790 | 1.07 | 0.08 | 1.000 |
| | *CXCR4* | 0.87 | 0.07 | 1.000 | 4.65 | 0.19 | <0.001 | 10.39 | 0.86 | <0.001 |
| | *VEGFA* | 1.21 | 0.03 | 0.282 | 1.90 | 0.12 | 0.038 | 2.30 | 0.11 | <0.001 |
| | *SNAI1* | 1.00 | 0.26 | 0.991 | 0.90 | 0.14 | 0.980 | 1.00 | 0.07 | 1.000 |
| | *SNAI2* | 0.69 | 0.18 | 0.504 | 1.46 | 0.07 | 0.002 | 1.69 | 0.12 | 0.002 |
| | *ZEB1* | # | | | # | | | # | | |
| | *ZEB2* | 1.76 | 0.42 | 1.000 | 2.62 | 0.48 | 0.314 | 3.73 | 0.73 | 0.043 |
| | *TWIST1* | # | | | # | | | # | | |
| | *CDH1* | 0.54 | 0.03 | 0.008 | 0.72 | 0.07 | 0.424 | 0.41 | 0.00 | <0.001 |
| | *KRT19* | 1.03 | 0.16 | 1.000 | 1.04 | 0.05 | 0.999 | 1.03 | 0.07 | 1.000 |
| | *OCLN* | 0.72 | 0.08 | 0.367 | 0.35 | 0.01 | 0.005 | 0.16 | 0.01 | <0.001 |
| | *STEAP1* | 1.33 | 0.11 | 0.124 | 0.85 | 0.07 | 0.602 | 1.43 | 0.08 | 0.015 |
| | *TSPAN13* | 0.85 | 0.03 | 0.246 | 0.61 | 0.05 | <0.001 | 0.51 | 0.02 | <0.001 |
| | *CDH2* | 0.64 | 0.03 | <0.001 | 0.95 | 0.05 | 1.000 | 0.34 | 0.02 | <0.001 |
| | *FN1* | 0.69 | 0.10 | 0.201 | 3.79 | 0.20 | <0.001 | 4.14 | 0.09 | <0.001 |
| | *ITGA5* | 0.94 | 0.08 | 0.989 | 4.64 | 0.13 | 0.004 | 8.21 | 0.31 | 0.001 |
| | *BMP2* | 0.69 | 0.06 | 0.013 | 1.92 | 0.18 | <0.001 | 1.90 | 0.08 | <0.001 |
| | *NID2* | 0.65 | 0.21 | 0.780 | 0.99 | 0.04 | 1.000 | 0.42 | 0.05 | 0.318 |
| | *SERPINE1* | 2.22 | 0.15 | <0.001 | 1.34 | 0.04 | 0.006 | 2.55 | 0.09 | <0.001 |
| | *DSC2* | 0.96 | 0.18 | 0.999 | 1.31 | 0.22 | 0.990 | 1.28 | 0.12 | 0.955 |
| | *DNMT1* | 0.77 | 0.04 | 0.037 | 0.60 | 0.04 | <0.001 | 0.40 | 0.01 | <0.001 |
| | *DNMT3A* | 1.22 | 0.11 | 0.507 | 1.00 | 0.06 | 1.000 | 0.92 | 0.02 | 1.000 |
| | *DNMT3B* | 0.80 | 0.04 | 0.037 | 0.48 | 0.02 | <0.001 | 0.32 | 0.01 | 0.018 |
| | *TET1* | # | | | # | | | # | | |
| **MIA PaCa-2** | *CCL5* | 7.07 | 0.45 | 0.023 | 0.33 | 0.03 | 0.999 | 2.95 | 0.22 | 0.091 |
| | *CXCR4* | 6.72 | 0.54 | <0.001 | 7.31 | 0.76 | <0.001 | 24.36 | 1.18 | <0.001 |
| | *VEGFA* | 1.44 | 0.53 | 1.000 | 1.89 | 0.46 | 1.000 | 2.56 | 0.62 | 0.617 |
| | *SNAI1* | 1.34 | 0.02 | 0.305 | 0.81 | 0.02 | 0.555 | 0.95 | 0.04 | 0.998 |
| | *SNAI2* | # | | | # | | | # | | |
| | *ZEB1* | 0.75 | 0.02 | 0.830 | 0.52 | 0.03 | 0.393 | 1.01 | 0.01 | 1.000 |
| | *ZEB2* | # | | | # | | | # | | |
| | *TWIST1* | # | | | # | | | # | | |
| | *CDH1* | # | | | # | | | # | | |
| | *KRT19* | 1.49 | 0.10 | 0.104 | 1.03 | 0.03 | 1.000 | 1.48 | 0.08 | 0.097 |
| | *OCLN* | 1.37 | 0.09 | 0.197 | 0.76 | 0.07 | 0.257 | 1.06 | 0.09 | 1.000 |
| | *STEAP1* | # | | | # | | | # | | |
| | *TSPAN13* | 1.21 | 0.12 | 1.000 | 0.65 | 0.05 | 0.169 | 0.72 | 0.08 | 0.328 |
| | *CDH2* | # | | | # | | | # | | |
| | *FN1* | 1.1 | 0.13 | 1.000 | 0.99 | 0.04 | 1.000 | 1.63 | 0.18 | 0.017 |
| | *ITGA5* | 1.9 | 0.25 | 1.000 | 0.96 | 0.15 | 0.235 | 1.8 | 0.19 | 1.000 |
| | *BMP2* | # | | | # | | | # | | |
| | *NID2* | # | | | # | | | # | | |
| | *SERPINE1* | 2.76 | 0.15 | <0.001 | 1.14 | 0.04 | 0.287 | 4.15 | 0.21 | <0.001 |
| | *DSC2* | # | | | # | | | # | | |
| | *DNMT1* | 0.74 | 0.03 | 1.000 | 0.54 | 0.01 | 1.000 | 0.56 | 0.02 | 1.000 |
| | *DNMT3A* | 0.96 | 0.03 | 1.000 | 0.49 | 0.02 | 0.013 | 0.65 | 0.10 | 0.421 |
| | *DNMT3B* | 1.01 | 0.03 | 1.000 | 0.51 | 0.03 | <0.001 | 0.51 | 0.04 | <0.001 |

| | | | | | | | | | | |
|---|---|---|---|---|---|---|---|---|---|---|
| | *TET1* | 1.96 | 0.24 | 0.011 | 1.01 | 0.22 | 0.995 | 3.11 | 0.27 | <0.001 |
| **PANC-1** | *CCL5* | 2.48 | 0.40 | 0.184 | 0.71 | 0.14 | 0.812 | 1.14 | 0.11 | 1.000 |
| | *CXCR4* | 2.26 | 0.47 | 1.000 | 10.74 | 0.66 | <0.001 | 25.61 | 1.99 | <0.001 |
| | *VEGFA* | 0.96 | 0.25 | 0.991 | 2.46 | 0.16 | 0.001 | 2.00 | 0.07 | 0.007 |
| | *SNAI1* | 0.98 | 0.02 | 1.000 | 0.77 | 0.05 | 0.685 | 0.12 | 0.01 | <0.001 |
| | *SNAI2* | 0.50 | 0.04 | 0.073 | 1.47 | 0.09 | 0.955 | 0.75 | 0.09 | 1.000 |
| | *ZEB1* | 1.35 | 0.06 | 0.037 | 0.85 | 0.05 | 0.401 | 0.73 | 0.02 | 0.026 |
| | *ZEB2* | # | | | # | | | # | | |
| | *TWIST1* | # | | | # | | | # | | |
| | *CDH1* | # | | | # | | | # | | |
| | *KRT19* | 1.15 | 0.27 | 1.000 | 1.02 | 0.03 | 1.000 | 1.13 | 0.09 | 0.980 |
| | *OCLN* | 1.03 | 0.26 | 1.000 | 0.96 | 0.25 | 0.994 | 0.96 | 0.12 | 1.000 |
| | *STEAP1* | # | | | # | | | # | | |
| | *TSPAN13* | 0.75 | 0.05 | 0.022 | 0.97 | 0.09 | 1.000 | 0.98 | 0.05 | 1.000 |
| | *CDH2* | # | | | # | | | # | | |
| | *FN1* | 0.61 | 0.13 | 0.430 | 2.02 | 0.17 | 0.002 | 1.03 | 0.05 | 1.000 |
| | *ITGA5* | 1.11 | 0.30 | 1.000 | 0.88 | 0.24 | 0.965 | 1.32 | 0.02 | 0.745 |
| | *BMP2* | # | | | # | | | # | | |
| | *NID2* | # | | | # | | | # | | |
| | *SERPINE1* | 0.97 | 0.06 | 1.000 | 0.67 | 0.05 | 0.015 | 0.83 | 0.07 | 0.234 |
| | *DSC2* | 1.51 | 0.33 | 0.899 | 2.05 | 0.27 | 0.294 | 5.76 | 0.27 | 0.006 |
| | *DNMT1* | 0.74 | 0.08 | 0.556 | 0.44 | 0.02 | 0.006 | 0.29 | 0.16 | 0.039 |
| | *DNMT3A* | 0.52 | 0.03 | 0.110 | 0.34 | 0.03 | 0.013 | 0.45 | 0.02 | 0.057 |
| | *DNMT3B* | 0.85 | 0.14 | 0.893 | 0.25 | 0.07 | 0.022 | 0.21 | 0.09 | 0.013 |
| | *TET1* | 1.46 | 0.09 | 0.007 | 1.26 | 0.09 | 0.140 | 0.41 | 0.02 | <0.001 |
| **SU.86.86** | *CCL5* | 0.75 | 0.02 | 0.024 | 1.47 | 0.13 | 0.006 | 1.60 | 0.12 | <0.001 |
| | *CXCR4* | 1.68 | 0.21 | 0.102 | 6.03 | 1.37 | 0.004 | 7.95 | 0.86 | <0.001 |
| | *VEGFA* | 2.81 | 0.50 | 0.015 | 6.07 | 1.03 | 0.037 | 18.91 | 0.66 | <0.001 |
| | *SNAI1* | 1.67 | 0.10 | 0.642 | 0.34 | 0.09 | 0.021 | 2.34 | 0.06 | 0.084 |
| | *SNAI2* | 0.69 | 0.13 | 0.397 | 2.65 | 0.21 | 0.001 | 2.32 | 0.15 | 0.004 |
| | *ZEB1* | # | | | # | | | # | | |
| | *ZEB2* | 1.02 | 0.19 | 1.000 | 0.86 | 0.07 | 1.000 | 1.77 | 0.17 | 0.069 |
| | *TWIST1* | # | | | # | | | # | | |
| | *CDH1* | 0.92 | 0.03 | 0.996 | 1.15 | 0.21 | 1.000 | 1.19 | 0.05 | 0.925 |
| | *KRT19* | 0.93 | 0.08 | 1.000 | 1.32 | 0.17 | 0.536 | 1.77 | 0.10 | 0.003 |
| | *OCLN* | 1.28 | 0.15 | 0.067 | 1.52 | 0.10 | 0.704 | 1.57 | 0.11 | 0.080 |
| | *STEAP1* | 1.32 | 0.30 | 1.000 | 0.52 | 0.09 | 0.352 | 0.88 | 0.20 | 0.990 |
| | *TSPAN13* | 0.94 | 0.16 | 1.000 | 0.38 | 0.03 | 0.087 | 0.50 | 0.12 | 0.331 |
| | *CDH2* | 1.22 | 0.10 | 0.425 | 0.64 | 0.20 | 1.000 | 1.47 | 0.10 | 0.013 |
| | *FN1* | 1.50 | 0.06 | 0.032 | 11.11 | 1.41 | <0.001 | 10.84 | 0.45 | <0.001 |
| | *ITGA5* | 1.50 | 0.28 | 1.000 | 4.13 | 1.31 | 0.266 | 17.70 | 0.98 | 0.003 |
| | *BMP2* | 1.47 | 0.07 | 0.614 | 1.60 | 0.25 | 0.449 | 3.34 | 0.05 | 0.006 |
| | *NID2* | 1.94 | 0.20 | 0.007 | 0.73 | 0.05 | 0.920 | 2.58 | 0.44 | 0.081 |
| | *SERPINE1* | 1.41 | 0.09 | 0.340 | 2.57 | 0.97 | 1.000 | 5.07 | 0.69 | <0.001 |
| | *DSC2* | 1.47 | 0.24 | 0.792 | 0.80 | 0.16 | 0.890 | 2.60 | 0.30 | 0.035 |
| | *DNMT1* | 0.90 | 0.07 | 0.733 | 0.54 | 0.03 | <0.001 | 0.47 | 0.01 | <0.001 |
| | *DNMT3A* | 0.91 | 0.03 | 0.722 | 0.63 | 0.08 | 0.064 | 0.77 | 0.02 | 0.050 |
| | *DNMT3B* | 0.90 | 0.04 | 0.978 | 0.58 | 0.04 | 0.001 | 0.48 | 0.01 | <0.001 |
| | *TET1* | 1.32 | 0.14 | 0.442 | 1.01 | 0.06 | 1.000 | 1.32 | 0.13 | 0.258 |

# Unexpressed in Controls (DMEM under normoxic conditions); CM, conditioned media in normoxia; HY, DMEM in hypoxia; CM+HY, CM in hypoxia

**Table S5.** DNA methylation and gene expression changes after DAC treatment

| | | DNA methylation | | | | | Gene expression | | |
|---|---|---|---|---|---|---|---|---|---|
| **Condition** | | **Control** | | **DAC** | | | **DAC** | | |
| **Cell line** | **Gene** | **%** | **SEM** | **%** | **SEM** | **p-value** | **FC** | **SEM** | **p-value** |
| **BxPC-3** | *TWIST1* | 77.43 | 0.53 | 57.66 | 1.83 | <0.001 | 99.2[#] | | |
| | *CDH1* | na | | na | | | 1.79 | 0.27 | 0.042 |
| | *KRT19* | na | | na | | | 0.48 | 0.05 | 0.001 |
| | *OCLN* | na | | na | | | 4.43 | 0.22 | <0.001 |
| | *STEAP1* | 53.33 | 5.17 | 49.88 | 4.27 | 0.38 | 1.14 | 0.11 | 0.339 |
| | *TSPAN13* | na | | na | | | 1.85 | 0.1 | <0.001 |
| | *CDH2* | 88.49 | 3.43 | 67.85 | 3.90 | 0.002 | 4.79 | 0.41 | 0.004 |
| | *FN1* | 7.57 | 2.02 | 9.47 | 3.01 | 0.494 | 4.55 | 0.14 | <0.001 |
| | *ITGA5* | na | | na | | | 3.42 | 0.12 | 0.008 |
| | *BMP2* | 4.44 | 1.26 | 4.55 | 1.35 | 1.000 | 3.98 | 0.37 | 0.003 |
| | *NID2* | 93.51 | 0.40 | 68.38 | 3.42 | <0.001 | 0.87 | 0.04 | 0.019 |
| | *DSC2* | 4.63 | 0.90 | 4.57 | 1.11 | 0.949 | 5.46 | 0.19 | 0.002 |
| **MIA PaCa-2** | *TWIST1* | 95.48 | 1.85 | 44.52 | 5.38 | <0.001 | 15.4[#] | | |
| | *CDH1* | na | | na | | | 68.5[#] | | |
| | *KRT19* | na | | na | | | 9.34 | 0.31 | <0.001 |
| | *OCLN* | na | | na | | | 1.56 | 0.11 | 0.005 |
| | *STEAP1* | 81.04 | 1.01 | 50.67 | 2.47 | 0.001 | 45.3[#] | | |
| | *TSPAN13* | na | | na | | | 7.97 | 0.16 | <0.001 |
| | *CDH2* | 97.57 | 0.29 | 49.42 | 0.79 | <0.001 | 356.5[#] | | |
| | *FN1* | 83.09 | 4.58 | 54.17 | 1.39 | <0.001 | 5.69 | 0.3 | <0.001 |
| | *ITGA5* | na | | na | | | 2.46 | 0.12 | 0.004 |
| | *BMP2* | 83.27 | 5.02 | 61.20 | 3.93 | 0.007 | 656. [#] | | |
| | *NID2* | 92.23 | 1.80 | 39.72 | 1.88 | <0.001 | 631.4[#] | | |
| | *DSC2* | 81.42 | 2.56 | 50.58 | 4.89 | 0.002 | 148.9[#] | | |
| **PANC-1** | *TWIST1* | 95.39 | 2.13 | 42.34 | 3.39 | <0.001 | 17.8[#] | | |
| | *CDH1* | na | | na | | | 106.4[#] | | |
| | *KRT19* | na | | na | | | 1.39 | 0.08 | 0.003 |
| | *OCLN* | na | | na | | | 1.66 | 0.07 | 0.001 |
| | *STEAP1* | 50.29 | 3.70 | 39.49 | 1.83 | 0.144 | 15.1[#] | | |
| | *TSPAN13* | na | | na | | | 2.36 | 0.23 | <0.001 |
| | *CDH2* | 94.95 | 0.26 | 48.55 | 5.39 | <0.001 | 282.1[#] | | |
| | *FN1* | 86.50 | 0.63 | 50.73 | 5.90 | <0.001 | 1.94 | 0.02 | 0.006 |
| | *ITGA5* | na | | na | | | 1.37 | 0.16 | 0.075 |
| | *BMP2* | 79.24 | 2.83 | 66.02 | 2.03 | 0.114 | 749.4[#] | | |
| | *NID2* | 87.42 | 0.04 | 45.13 | 6.61 | <0.001 | 174.8[#] | | |
| | *DSC2* | 36.27 | 3.33 | 30.71 | 2.49 | 0.040 | 46.85 | 11.15 | <0.001 |
| **SU.86.86** | *TWIST1* | 95.07 | 2.99 | 74.15 | 3.69 | <0.001 | 332.8[#] | | |
| | *CDH1* | na | | na | | | 1.52 | 0.13 | 0.004 |
| | *KRT19* | na | | na | | | 0.59 | 0.04 | <0.001 |
| | *OCLN* | na | | na | | | 2.32 | 0.21 | 0.002 |
| | *STEAP1* | 59.15 | 0.85 | 57.75 | 0.19 | 0.723 | 1.13 | 0.08 | 0.390 |
| | *TSPAN13* | na | | na | | | 0.79 | 0.04 | 0.043 |
| | *CDH2* | 4.08 | 0.92 | 4.30 | 1.35 | 0.886 | 2.42 | 0.16 | <0.001 |

| | | | | | | | | |
|---|---|---|---|---|---|---|---|---|
| *FN1* | 6.80 | 1.51 | 5.93 | 0.29 | 0.717 | 10.23 | 0.69 | 0.029 |
| *ITGA5* | na | | na | | | 3.97 | 0.2 | <0.001 |
| *BMP2* | 3.50 | 0.02 | 4.54 | 1.58 | 0.478 | 2.93 | 0.21 | 0.016 |
| *NID2* | 36.55 | 2.07 | 35.69 | 4.11 | 0.715 | 6.5 | 0.39 | <0.001 |
| *DSC2* | 5.12 | 1.42 | 6.38 | 2.63 | 0.442 | 2.65 | 0.18 | 0.002 |

na, not analyzed; # Unexpressed in Controls, Fold Change calculated based on ct = 40
